# Reporting accuracy of packed lunch consumption among Danish 11-year-olds differ by gender

**DOI:** 10.3402/fnr.v57i0.19621

**Published:** 2013-03-06

**Authors:** Nina Lyng, Sisse Fagt, Michael Davidsen, Camilla Hoppe, Bjørn Holstein, Inge Tetens

**Affiliations:** 1 Division of Nutrition, National Food Institute, Technical University of Denmark, Kongens Lyngby, Denmark; 2 National Institute of Public Health, University of Southern Denmark, Odense, Denmark

**Keywords:** school lunch, self-reported intake, recall accuracy

## Abstract

**Background:**

Packed lunch is the dominant lunch format in many countries including Denmark. School lunch is consumed unsupervised, and self-reported recalls are appropriate in the school setting. However, little is known about the accuracy of recalls in relation to packed lunch.

**Objective:**

To assess the qualitative recall accuracy of self-reported consumption of packed lunch among Danish 11-year-old children in relation to gender and dietary assessment method.

**Design:**

A cross-sectional dietary recall study of packed lunch consumption. Digital images (DIs) served as an objective reference method to determine food items consumed. Recalls were collected with a lunch recall questionnaire (LRQ) comprising an open-ended recall (OE-Q) and a pre-coded food group prompted recall (PC-Q). Individual interviews (INTs) were conducted successively. The number of food items was identified and accuracy was calculated as match rates (% identified by DIs and reported correctly) and intrusion rates (% not identified by DIs but reported) were determined.

**Setting and subjects:**

Three Danish public schools from Copenhagen. A total of 114 Danish 11-year-old children, mean (SE) age=11.1 (0.03), and body mass index=18.2 (0.26).

**Results:**

The reference (DIs) showed that girls consumed a higher number of food items than boys [mean (SE) 5.4 (0.25) vs. 4.6 (0.29) items (*p*=0.05)]. The number of food items recalled differed between genders with OE-Q recalls (*p*=0.005) only. Girls’ interview recalls were more accurate than boys’ with higher match rates (*p*=0.04) and lower intrusion rates (*p*=0.05). Match rates ranged from 67–90% and intrusion rates ranged from 13–39% with little differences between girls and boys using the OE-Q and PC-Q methods.

**Conclusion:**

Dietary recall validation studies should not only consider match rates as an account of accuracy. Intrusions contribute to over-reporting in non-validation studies, and future studies should address recall accuracy and inaccuracies in relation to gender and recall method.

From a nutrition and public health perspective, it is important to develop valid methods for assessing children's self-reported consumption during school hours. The school setting is often accentuated as an appropriate setting for health-promoting interventions (1) and a large proportion of the daily food intake of school aged children is consumed in this extra-familiar context (2). Measurement of food consumption in the school setting poses methodological challenges because food is consumed relatively unsupervised either in the classroom or in the canteen. Parents and school meal providers may have exact knowledge about the food served but since they are not present during the meal they cannot be expected to provide accurate reports on behalf of their children.

Packed lunch is the more prevalent lunch format in several countries, including Denmark (3–5). In Denmark, packed lunch usually consists of open sandwiches on rye bread with cold cuts and supplementary vegetables and fruits. Data from the Danish National Survey of Dietary Habits and Physical Activity 2005–2008. 93% of children aged 7–10-year-old and 83% of children aged 11–15-year-old consumed lunch at least six times per week (Christensen, 2012, unpublished data). A recent Danish study showed that parents primarily prepared packed lunches and children expressed satisfaction with the content of the lunch packages (6).

Several studies have assessed the nutritional content of packed lunches either by using digital images (DIs) (7), direct observation (8), or weighing food served and plate waste as a means of assessing energy distribution and nutritional content (9). A recent Danish study suggested that packed lunches contained more saturated fat compared to school lunches, on average (10).

One well acknowledged methodological challenge in dietary assessment methods is that accuracy of self-reported consumption may differ between genders in populations of children. Gender has been shown to be associated with diet-related outcomes in terms of actual consumption (11–13) and meal pattern (14). In relation to fruit and vegetable consumption, it has been argued that girls have greater knowledge and self-efficacy compared to boys, although these determinants did not explain the gender differences in intake after adjusting for preferences and perceived accessibility (12).

In previous studies, diet-related self-reported outcomes have identified gender-specific differences in groups of children's in terms of dropout rate (15), under-estimation of portion sizes (16), and body weight (17, 18). In addition to the individual characteristics, a range of design factors may influence the accuracy of recalls, for example, retention period, interview format, target period, and interview time (8, 19, 20).

Conceptually, accuracy can be divided into *qualitative accuracy*, which is accuracy at the food level, and *quantitative accuracy*, which concerns the aspects of quantifying amounts, consumed (21). Validation studies of children's self-reported lunch intake in which recall accuracy is addressed, qualitatively distinguish between *matches* (food items reported and observed by an objective measure), *omissions* (food items not reported but observed by an objective measure) and *intrusions* (food items reported but not observed by an objective measure) (22, 23). Direct observation is a valid objective method known to be successfully applied in school settings (19, 24–26), but the method is expensive and time consuming and thus difficult to apply in a population-based setting (27, 28).

The methodological aspect of accuracy in relation to self-reported school hour consumption is understudied and needs further exploration. Existing knowledge about reporting accuracy of school hour consumption obtained by self-administered questionnaires among schoolchildren is limited (4, 15, 29). Most of the identified validation studies employ interviews as a primary self-reported data collection method (4, 30, 31). As an alternative to direct observation, a growing number of studies apply DIs as the objective measure of actual consumption in school settings (32–34). However, the studies have not been conducted in relation to recall accuracy.

Little is known about the association between gender and recall accuracy among school aged children. The existing studies have been conducted in relation to school meals in which gender did not influence omission rates or intrusion rates (30). However, the association in relation to packed lunch is understudied, and it has been argued that it is difficult to identify the content of packed lunches using observation as a validation method (30, 31).

The objective of the present study was to assess qualitative accuracy of self-reported packed lunch consumption obtained by questionnaires and interviews among Danish 11-year-olds in relation to gender.

## Material and methods

### Setting and design

The study was a cross-sectional study with 114 Danish 5th grade students from three public schools in Copenhagen [mean age=11.1, standard error (SE)=0.01]. Five schools with the highest participation rate in the School Lunch Scheme EAT (35) were identified by the Children and Youth Administration, Municipality of Copenhagen, and were invited to participate. Three schools accepted the invitation, and all 5th grade students received a written invitation including a parental consent form. A total of 205 students were invited of which 189 were present on the day of data collection. Children who did not consume lunch were excluded (*n*=5) and children from whom we were not able to obtain complete data were also excluded which resulted in a 67% response rate. Assent was collected from the children before participation. The project was approved by the Danish Data Protection Agency prior to initiation of the data collection.

The target period was self-reported same day intake and prompting was forward ordered ranging from morning to lunch, although only lunch intake was assessed against the objective reference. Lunch intake data was collected immediately after the lunch break, which kept the total retention period under a maximum of 1½ hours.

The lunch recall questionnaire (LRQ) consisted of an open-ended part (OE-Q) and a pre-coded part (PC-Q) and individual face-to-face interviews (INTs). The LRQ was completed prior to the interviews, as our primary focus was to test accuracy for recalls obtained by the questionnaires. DIs of lunch consumption were included as an objective reference against which self-reported recalls were assessed. Lunch consumption data were complemented with self-reported information about age and gender, and objective anthropometric measures.

### Digital images

DIs were chosen as the objective reference. The images served to identify food items and assess actual intake by comparison of a corresponding set of pre-meal and post-meal images. Members of the research team photographed students’ packed lunches using a validated standard protocol as described previously (10).

The pre-meal image was taken prior to consumption and therefore showed all food items served on the plate. Students were instructed to unpack their lunch and place all foods on a plate with their unique identification number. Further, they were instructed to lift cold cuts and sandwich fillings before the image was taken to ensure subsequent identification of, for example, fat spreads. The post-meal image was taken following consumption. The post-meal image displayed an empty plate for those who had eaten everything and plate waste in case the child had left overs. Nikon Coolpix S210 cameras with electronic Vibration Reduction stabilization and motion detection were used and images were taken using a Cubelite kit from Lastolite.

### Food-based non-quantitative questionnaire (LRQ)

A self-administered LRQ was developed for the purpose and reports were restricted to the food level. The questionnaire contained the following two self-reported measures: *open-ended* (OE-Q) where students were instructed to write down everything they had consumed for lunch and *pre-coded* (PC-Q) in which self-reports were prompted by pre-coded food groups. Self-reported lunch consumption was obtained with a lunch recall questionnaire (LRQ) developed and pre-tested on 50 11-year-olds from a school situated in Copenhagen.

Food groups and food items were selected based on knowledge of lunch intake in the particular age group from the representative National Survey of Dietary Habits and Physical Activity (DANSDA) (36, 37) and Guidelines for healthy meals in Schools and Kindergartens (2, 36). The LRQ was administered in the classroom, and students completed them individually, immediately after consumption or after the adjacent lunch break. Time to complete the questionnaire ranged from 5–15 min, and variation was mainly due to the different number of food items consumed.

### Individual interviews

Individual face-to-face interviews were conducted by trained interviewers right after the child had completed the questionnaire. Interviews (INTs) followed a multi pass protocol as described in Baxter et al. (22). Initially students were asked to recall everything they had consumed for lunch, followed by a non-directive prompt, and finally recalls were prompted by food groups. Interviews were conducted at a quiet location at the school. Duration of the interviews ranged from 4–8 min. All interviews were recorded (Olympus WS-450S digital voice recorder) and subsequently food level recalls were transcribed.

### Anthropometrics

Students were measured and weighed by a member of the research team after completion of the self-reported methods, that is, after lunch consumption. Height was measured to the nearest centimeter (Soenhle 5003.01.001), and weight was measured in kilograms, with one decimal (OBH Nordica, personal scale), according to the standard protocol of Division of Nutrition; that is, students were measured without shoes and both height and weight were measured twice (Fagt, 2012 personal communication). Gender- and age-specific cutoffs were employed to assess body mass index (BMI) (38).

### Intake variables and assessment of recall accuracy

The specific food items included in the LRQ were grouped into six food groups [i.e. bread, fat spreads, cold cuts, fruit including nuts (39), vegetables, and snacks] containing a total of 18 subgroups and single food items. Consumption of food items obtained by DIs, OE-Q, PC-Q, and INTs were identified and characterized according to the pre-determined food groups. Actual intake was assessed by comparing the corresponding set of images. Accuracy was described as match rates and intrusion rates and was estimated in two steps. First, all food items were identified as matches, omissions, and intrusions by comparing the objectively determined food items with the self-reported consumption by the OE-Q, PC-Q, and INTs as previously described (22, 23). Second, recall accuracy for the OE-Q, PC-Q, or INTs was assessed by calculating match rates and intrusion rates in the following way:

Match rate=∑matches/(∑matches+∑omissions)*100.

Intrusion rate=∑intrusion/(∑matches+∑intrusions)*100.

### Statistics

Characteristics of the study population were stratified by gender and presented as means with SE. Number of food items consumed obtained by the objective reference and the self-reported measures were stratified by gender and the Kruskal–Wallis test was applied. Finally, matched *t*-tests were conducted to compare the mean number of food items identified by both DIs and self-reports and to determine which self-reported method was more accurate, i.e. to compare match rates and intrusion rates between methods. Statistical analyses were conducted with SAS (version 9.2 for windows, SAS Institute Inc., Cary, NC, USA).

## Results

The study population characteristics are shown in Table 1. The two sample *t*-tests did not show significant differences between boys and girls in BMI distribution. Successive analyses were not stratified by BMI.

**Table 1:** Characteristics of the study population (*N*=114)

	Girls (*n*=65)		Boys (*n*=49)		
			
	Mean	SE	Mean	SE	*p* *
Age (years)	11.1	0.35	11.1	0.39	0.40
Height (m)	1.51	0.01	1.51	0.01	0.83
Weight (kg)	40.4	1.00	44.0	1.19	0.09
BMI (kg/m^2^)	17.7	0.29	19.0	0.38	0.07

* Two sample *t*-test for difference in mean.

Table 2 shows the number of food items consumed according to the objective reference (DIs) and the three self-reported measures: OE-Q, PC-Q, and INTs. Stratification by gender showed that girls consumed significantly more food items (5.4) compared to boys (4.6) (*p*=0.05) when consumption was determined by DIs. Food consumption reported by OE-Q showed significant differences in the mean number of food items reported and girls reported significantly more food items (4.2) than boys (3.3) (*p*=0.005). Self-reports obtained by PC-Q and INTs did not differ significantly by gender.

**Table 2:** Number of food items obtained by digital images (DIs) and self-reported methods: Open-ended questionnaire part (OE-Q), pre-coded questionnaire part (PC-Q), and interviews (INTs)

	Girls (*n=*65)		Boys (*n=*49)		
			
Method	Mean	SE	Mean	SE	*p* *
DIs	5.4^a^	0.25	4.6^a^	0.30	0.05
OE-Q	4.2^b^	0.22	3.3^b^	0.22	0.005
PC-Q	5.6^a^	0.29	5.1^a^	0.30	0.29
INTs	5.6^a^	0.24	5.1^a^	0.25	0.06

Packed lunch consumption in 11-year-old children (*N*=114).

* Kruskal–Wallis test for gender difference. Different superscript letters a and b in each column show significantly different rates (*p*<0.001) when comparing self-reported methods with DIs. Matched *t*-test.

Match rates and intrusion rates for recalls obtained by the self-reported measures stratified by gender are presented in Table 3. Gender-specific differences were shown for recalls obtained by INTs where girls’ match rate was significantly higher (89.7% vs. 84.4%), and girls’ intrusion rate was significantly lower compared to the corresponding rates for boys (14.6% vs. 23.3%) (*p*=0.04 and *p*=0.05, respectively).

**Table 3:** Match rates and intrusion rates by three self-reported methods: Open-ended questionnaire part (OE-Q), pre-coded questionnaire part (PC-Q), and interviews (INTs)

		Girls (*n*=65)		Boys (*n*=49)		
				
Rates (%)	Self-reported method	Mean	SE	Mean	SE	*p* ‡
Match rate*	OE-Q	70.8^b^	2.85	65.4^b^	3.96	0.24
	PC-Q	73.8^b^	2.83	71.5^b^	3.70	0.62
	INTs	89.7^a^	1.95	84.4^a^	2.40	0.04
Intrusion rate†	OE-Q	11.8^d^	2.14	12.8^e^	2.51	0.70
	PC-Q	27.2^e^	2.86	35.9^c^	3.76	0.09
	INTs	14.6^d^	2.17	23.3^d^	3.41	0.05

Packed lunch consumption in 11-year-old children (*N=*114).

* Match rate=Σmatches/(Σmatches+Σomissions)×100.

† Intrusion rate=Σintrusions/(Σmatches+Σintrusions)×100.

‡ Kruskal–Wallis test for gender difference. Match rate and intrusion rate: Different superscript letters a–e in each column show significantly different (*p*<0.01) rates when comparing self-reported methods. Gender- and age-specific cutoffs were employed to assess body mass index (38). Matched *t*-test.

Comparisons between the self-reported methods showed that INTs provided match rates that were significantly higher compared with self-reports from the questionnaire methods OE-Q and PC-Q. The corresponding comparisons for intrusion rates were more varied. Intrusion rates were highest for recalls obtained with PC-Q irrespective of gender.

## Discussion

The study provides insight into the unexplored subject of accuracy of packed lunch recalls and pointed at several gender-specific differences in actual consumption, response behavior, and recall accuracy. Girls consumed more food items than boys as determined by DIs, and girls reported significantly more food items with OE-Q compared with boys. In addition, recalls obtained by INTs showed that girls’ recalls were more accurate both in terms of a higher match rate and a lower intrusion rate.

### Gender issues

Gender-specific differences in consumption have been shown previously in relation to energy intake (40). However, as has been shown in an earlier study by Baxter et al., nutrient level analyses do not necessarily reveal differences in consumption at the food level and inaccurate recalls at the food level may provide accurate nutrient level analyses (30). Extensive knowledge of which food groups and food items are correctly (matches) and incorrectly (intrusions) reported can inform future advances in the methodology of self-reported recalls. The result that girls consumed more different food items than boys has been shown in relation to school meals (11), and overall it may indicate that girls consume a more varied lunch compared with boys.

Girls reported more food items with all three self-reported measures than boys although the difference was only significant for OE-Q. The result points at three potential explanations for the difference: a) consumption pattern differs between genders as discussed above or b) boys provide less accurate written recalls when reports are not prompted, or c) boys reports are less accurate compared with girls’ when obtained by interviews. Additional analyses could identify which food groups contribute to the variation and explore if the variations contribute to differences in diet quality. In such cases, inclusion of important determinants that mediate the differences in consumption, for example, preferences or perceived accessibility (12), should be taken into account in future studies.

The result that boys report fewer food items than girls with open-ended random-order written recalls is in concordance with results from the Danish sample of the Pro Children study (Krolner, personal communication). Consequently, prompting may be a feasible strategy to even out the difference in response behavior, although careful consideration regarding selection of prompting method is recommended (41, 42).

Inclusion of the objective method enabled us to distinguish between the explanations. As the reference showed a higher number of food items consumed, we would expect girls to report a higher number. The fact that girls’ recalls were more accurate with INTs may be explained by the fact that girls possessed a greater knowledge about foods as they are more likely to participate in meal preparations and food purchases than boys (12, 13).

### Recall methods

The study indicated interesting findings regarding the methods. The interview method provided the highest match rates and lowest intrusion rates. However, as a consequence of the fixed order of self-reported methods, we were not able to determine whether the higher accuracy for reports obtained by INTs was a consequence of learning effect or an expression of gender differences. Match rates ranged from 84–90% and similar high rates have been shown with same day recalls where retention period was restricted to a minimum of 90 min (30). The method is useful in small-scale studies, but less feasible among larger populations.

LRQ did not differ in the obtained match rates, but the intrusion rate was significantly higher for PC-Q. The high intrusion rate may be explained by the fact that recalls were prompted by food groups. In contrast, OE-Q children had to categorize single food items into pre-defined food groups. The food groups may have been similar or dissimilar to the children's own retrieval cues and consequently may have influenced recalls negatively (30) by prompting them to report food items not actually consumed or verified by the DIs. However, the OE-Q recalls were subjected to some degree of under-reporting because the number of food items was significantly lower than the number determined by the objective reference images. The advantages and disadvantages of OE-Q vs. PC-Q reports need further exploration to develop methods that are applicable with large samples of children in their natural contexts.

### Limitation and strengths

An inherent limitation the study was the lack of independency between the self-reported methods. The logistics and timely constraints in the school settings only allowed us to collect data on the same individual with the different methods on the same day. At the same time, the number of pupils was limited, and consequently the methods were administered in the same way for all pupils. In the analyses, we took the correlation between methods into account by choosing paired *t*-test statistics to assess the differences in accuracy measures.

Only children with a complete set of DIs, a completed OE-Q and PC-Q, who had also participated in the INTs, including objective anthropometric measurements, were included in the sample. The analytic sample consisted of 67% of the potential sample, and it is possible that non-participants differed in their ability to report their intake as pointed out by Berg (15). Other factors, for example, motivation to comply with the different methods, may also have influenced the participation rate.

Another limitation of the present study lies in the fact that the objective measures were only included for lunch. Consequently, the availability may have inflated intrusion rates because any pre-lunch consumption of food items from the packed lunch that might occur during the morning break would be classified as intrusions when reported by the children. The problem of pre-lunch consumption of packed lunch has been handled in non-validated studies in which pre-meal images have been taken in the beginning of the school day (40, 43). Another potential limitation was that the images may have served as a positive visual-prompting aid and could thereby have improved all match rates from all three self-reported methods, as well as questionnaire reports may have contributed to the high match rates obtained with the interview method. The potential limitations of inclusion of DIs as objective reference method were outweighed by the fact that the DIs provided a feasible validation method to study the understudied topic of reporting accuracy in relation to self-reported packed consumption among school children. The method provided a quick review of the content of the packed lunches at the food level where the qualitative accuracy could be determined.

### Implications

The study assessed the important aspect of qualitative reporting accuracy, but other aspects regarding the design of an optimal dietary assessment method for public health nutrition purposes call for considerations. This study pointed at two aspects that need further exploration. The first aspect concerns the relation between food served and food consumed in packed lunches. Although our study did not result in significant differences in the number of food items served, this aspect could be addressed in future studies in larger samples. If this is the case then future health-promoting activities could address this with parents and other caregivers that prepare lunch packages (6). The second aspect refers to the methodological question of ensuring that self-reported methods do not introduce differential reporting bias insofar that the methods appeal more to girls and may render boys’ reporting less accurately.

## Conclusion

Gender differences were expressed in relation to reporting accuracy and response behavior and in a variety of food numbers consumed. Girls’ self-reports were more accurate compared to boys’ with all self-reported measures although the difference was only significant for INTs. Self-reports obtained by OE-Q were subjected to under-reporting, and both genders reported significantly fewer items compared to DIs. Self-reports were subjected to both omissions and intrusions and demonstrated that further advances are recommended to improve self-reported school hour consumption.
